# Cross-sectional study on the epidemiological investigation ability of professional staff from Centers for Disease Control and Prevention in Guizhou Province

**DOI:** 10.3389/fpubh.2024.1335553

**Published:** 2024-05-20

**Authors:** He Huang, Guang-hai Yao, Jiao Huang, Bin Deng, Jun Wu, Chun Yu

**Affiliations:** ^1^Institution of Communicable Disease Prevention and Control, Guizhou Center for Disease Prevention and Control, Guiyang, China; ^2^Office for Disease Control and Emergence Response (Information Center), Guizhou Center for Disease Prevention and Control, Guiyang, China

**Keywords:** cross-sectional study, epidemiological investigator, professional staff, investigation ability, questionnaire

## Abstract

**Introduction:**

This study aims to evaluate the qualifications and identify skill enhancement areas for epidemiological investigators in Centers for Disease Control and Prevention (CDCs) in Guizhou’s, informing future training and policy initiatives to strengthen public health responses.

**Methods:**

A cross-sectional survey was conducted in August 2022, and an online, self-designed questionnaire on the Epidemiological Dynamic Data Collection platform was administered to evaluate the professional staff in CDCs. The responses were scored and presented using descriptive statistical methods, and the factors influencing the total score were analyzed by one-way ANOVA and linear regression.

**Results and discussion:**

A total of 1321 questionnaires were collected, yielding an average score of 14.86±3.49 and a qualification rate of 29.9%. The scoring rate of ability of individual protection and coordination in epidemic control was high (87.25%). Meanwhile, improvements in further training were needed in areas such as data analysis ability (23.67%), knowledge of site disinfection (40.40%), and epidemiological investigation skills (42.50%). No significant difference was observed between the scores of city and county CDCs, (*t* = 1.071, *p* =0.284). The effects of gender and age could be disregarded, and the experience in epidemiological work and training (including investigation on COVID-19 cases and contacts), educational background, and professional title partially explained the survey outcome (R Square of the linear regression model was 0.351). The survey indicated the need for additional well-trained epidemiologic investigators in Guizhou. Specified training was effective in improving epidemiologic investigation, and enhancement in data analysis ability and knowledge of field disinfection are recommended in professional staff cultivation.

## 1 Introduction

COVID-19 has become a global disaster from 2019 to 2022, resulting in over 596 million cases and 6.4 million deaths (1, 2). In the long and arduous fight against the virus, the epidemiological investigation is critical in identifying the source of infection, locating areas where strict control measures should be taken, and giving support to the government when and how to adjust the prevention and control policies. The ability to cope with emergencies is important during a confirmed outbreak. The demand for epidemiologists specializing in COVID-19 response has enormously increased worldwide (3–5). A rapid reaction at the early stage of an epidemic featuring the judgments of living areas of patients, close contact at each site, and the strict implementation of measures would turn the battle against COVID-19 into a controllable mode. The quality of the epidemiological investigation has a direct influence on the scope of disease control measures and the risk of epidemic spread. Therefore, the group of qualified epidemiological investigators has great significance in epidemic control.

In China, the epidemiological investigation of COVID-19 was conducted by the local Public Security Department, the Industry and Information Technology Department, and public health organizations, mainly consisting of the local Center for Disease Prevention and Control (CDC) and basic healthcare centers (6). The Public Security and Industry and Information Technology departments used their technologies and authorized security information systems to simultaneously locate the patients and the people exposed to different sites. The staff of CDCs and basic healthcare centers were adept at distinguishing the close contacts apart from other people on the sites based on their medical knowledge and the standard Prevention and Control Guidelines for COVID-19. Assessing the ability of CDC staff would help find their restrictions and weaknesses in epidemiological investigation and improve their efficiency and accuracy in further work.

The Guizhou Province is a mountainous area of multiethnicity in southwest China. A complicated geographic and social environment makes it difficult to classify people and their close contacts; hence, tracking depends more on modern technology and information systems. Traditional means, such as face-to-face investigation and telephone surveys, would provide helpful information. Therefore, the CDC staff should take an active role in epidemiological investigation. This survey aimed to determine how many epidemiology investigators in CDCs in Guizhou are qualified and which of their skills must be improved (7).

## 2 Methods

### 2.1 Subjects and survey platform

A cross-sectional study was conducted among professional staff in all municipal and prefectural CDCs in Guizhou Province from 15 July 2022 to 30 August 2022. The CDCs’ professional staff who were involved in or were prepared for epidemiological investigation on COVID-19 from 2020 to 2022 were invited to fill in an online questionnaire. A list of the number of epidemiological investigators in all municipal and prefectural CDCs was used to check the sample size. Three rounds of follow-ups were conducted by the leader in charge of the Provincial CDC to remind the CDCs with low response rates. The survey ended when the sample size reached 95% of the sum in the list.

The Epidemiological Dynamic Data Collection platform (EDDC) was developed independently by the Information Center of the Chinese CDC to collect epidemiological data on a mobile terminal and facilitate multicenter studies. Users were classified into different levels and were entitled to manage low-level user permissions and maintain data. Our research group was authorized to use the EDDC by the Chinese CDC to collect information among the professional staff in CDCs from Guizhou Province.

### 2.2 Questionnaire

The questionnaire contained eight parts, namely, demographic information, experience in handling the COVID-19 epidemic, professional training experience, ability of epidemiological investigation, disinfection skill, information analysis skill, knowledge of discovery and report of COVID-19, and individual protection and coordination experience. Every part consisted of three to seven related questions that were discussed by professional epidemiological investigation workers from the provincial CDC in Guizhou. Apart from demographic information, experience in the investigation of a COVID-19 case and professional training, the remaining five parts indicated abilities in epidemiological investigation The questionnaire is available in Supplementary Data Sheet 1.

Three questions focused on the experience in handling the COVID-19 epidemic, and each was scored with 0 to 1 point. Multilevel choices were scored as 0.2, 0.4, or 0.6 (listed in Table 1). Three items were dedicated to the experience in handling the COVID-19 epidemic, i.e., whether the subjects had participated in the epidemiological investigation, times of local COVID-19 response, and numbers of COVID-19-related epidemiological works in which they had been involved.

**Table 1:** Scores of experience handling the COVID-19 epidemic and the proportion of the points by item.

Items	Choices	Score	Account	Percentage%
Involved in COVID-19 emergency response	Yes	1	1,219	92.28
	No	0	102	7.72
Times of local COVID-19 response	None	0	109	8.25
	1 to 5	0.25	431	32.63
	6 to 10	0.5	113	8.55
	11 to 20	0.75	333	25.21
	Above 20	1	335	25.36
Kinds of work^a^ related to emergency response for the COVID-19 epidemic	No attendance	0	49	3.71
	One unrelated work	0.2	95	7.19
	One related work	0.5	395	29.9
	Two kinds of related works	0.7	387	29.3
	Three kinds of related works	0.9	232	17.56
	Over three kinds of related works	1	163	12.34

a Works related to emergency response to the COVID-19 epidemic include epidemiological investigation, field disinfection, joint investigation letter handling, epidemic information submission, nucleic acid sample collection, nucleic acid detection and quarantine site management.

Professional training programs for epidemiological investigation were conducted by CDCs from district to national levels. The training level was defined as the highest level that the subject had taken, and the level was coded from 1 to 4 points for district level, city level, provincial level, and national level. For example, if someone had been involved in the training programs held by the provincial CDC and municipal CDC, respectively, his or her training level was considered as provincial level, coded 3 points.

Training effect was defined as the individual assessment of training. If the subject chooses one positive attitude toward training, then the score adds 1 point. Otherwise, the score was −1 point. The sum of the five choices’ scores was the code of training effect. The core of professional training level added to the score of training effect was the score of professional training experience.

The ability to conduct the epidemiological investigation was defined as a comprehensive concept with five parts, namely, skills for epidemiological investigation, knowledge of site disinfection, self-evaluated data analysis ability, knowledge of case reports of COVID-19, and individual protection and coordination experience. In consideration of importance and the number of items in the content, the question about the content of the epidemiological report that the subject had ever written was set at 2 points, which was in the part of skills for epidemiological investigation. Other questions in five parts were scored 1 point. The subject was required to rate their data analysis report on a scale of 10 points, and the self-rated scale was then divided by 10, translating into a 1-point item.

The full score of a part varied from 3 to 10 points. Five parts’ scores were summed to indicate the individual’s ability to conduct an epidemiological investigation. The score and proportion of each question in the five sections are listed in the Supplementary Table 1. The scores of experiences in handling the COVID-19 epidemic and professional training acted as two predictors of the individual’s ability to conduct epidemiological investigations.

The core information consisted of structured data about a positive case of COVID-19 and included demographic information, nucleic acid test results, deduction of transmission chain, and close contacts. Core information is required in case reports to enable quick responses during an epidemic. Details were added in the epidemiological report with further investigations.

A pre-survey of 45 people was conducted among the trainees of a field epidemiology program at the Provincial CDC in Guizhou before the online survey. Some problems, such as ambiguity and lack of clarity in expression and redundant or inaccurate definitions, were proposed and modified in the latter edition of an online questionnaire. Logical errors were found, and some restrictions were set in the choices within different questions when the questionnaire was uploaded to EDDC.

### 2.3 Data collection and statistical analysis

After informed consent was obtained, the subjects answered the questionnaire on the EDDC for information collection and exportation. The data were exported and saved as a Microsoft Excel file on 30 August 2022. Missing data and some simple logical errors were avoided in the questionnaire design step on EDDC. If any problems with the data emerged, the researchers then confirmed the answers by calling back the subjects. The population of each prefecture was extracted from the basic information platform of the Chinese CDC. The number of epidemiological investigators serving per 100,000 people per prefecture was calculated by the following formula: number of epidemiological investigators for a prefecture/population of the prefecture × 100,000 = number of epidemiological investigators serving per 100,000 people per prefecture. The weighted mean score of a part was calculated by adding all average scores of questions belonging to a part. The scoring rate was the proportion of the weighted mean score, taking in the full score of a part.

Data analysis was conducted on IBM SPSS Statistics 27. One-way ANOVA was used to compare the differences within groups classified by factors and to filter the factors included in the regression model. Linear regression model was applied to analyzed the influencing factors of the score of epidemiological investigation ability.

## 3 Results

The target population comprised 88 county-level CDCs and nine city-level CDCs in Guizhou Province. A total of 1,321 questionnaires were collected, of which 198 were from city-level CDCs and 1,123 from county-level centers. The number of epidemiological investigators varied from 3 to 42 per prefecture, and the average was 12.45 ± 6.56. For the index of the number of epidemiological investigators serving per 100,000 people in a prefecture, the range was from 0.39 to 10.82, with an average of 3.75 ± 2.23. The demographic information of the staff is listed in Table 2.

**Table 2:** Demographic information of epidemiological investigators in Centers for Disease Control in Guizhou.

Items		Counts	Percentage%
Gender	Male	507	38.38
	Female	814	61.62
Age	20-	278	21.04
	30-	463	35.05
	40-	419	31.72
	50-	161	12.19
Education background	PhD^a^	1	0.08
	MD	45	3.41
	BD	870	65.86
	Below BD	405	30.65
Professional title	Professor	12	0.91
	Associate professor	122	9.24
	Doctor in charge	347	26.27
	Doctor	449	33.99
	None	391	29.6
Duration of epidemiological investigation	Less than 1 year	52	3.94
	1 to 5 years	894	67.68
	6 to 10 years	161	12.19
	11 to 20 years	133	10.07
	21 to 30 years	61	4.62
	More than 31 years	20	1.51
Specialization (multiple choices)	Prevention and control of infectious diseases	686	51.9
	Prevention and control of chronic diseases	198	14.99
	Immunization program	196	14.84
	Emergency management	165	12.49
	Prevention and control of AIDS	138	10.45
	Prevention and control of tuberculosis	118	8.93
	Prevention and control of occupational disease	115	8.71

a PhD is the abbreviation for Doctor of Philosophy, BD for bachelor’s degree, and MD for master’s degree.

### 3.1 Experience in handling the COVID-19 epidemic

From 1 January 2020 to 31 August 2022, 1,219 subjects self-reported to be involved in handling COVID-19 outbreaks, accounting for 92.28%. Among them, 1,133 participated in the epidemiological investigation, 613 in the processing of cooperative investigation letters, 264 in nucleic acid collection, 257 in information submission, 174 in field disinfection, and 171 in the management of isolation sites. The scores of experience in handling the COVID-19 epidemic are shown in Table 1.

### 3.2 Professional training experience

A total of 1,296 people, or 98.11%, had participated in training on COVID-19 prevention and control since the questionnaire survey started. Among them, 348 participated in the training at the district level, accounting for 26.34%, and 557 people participated in provincial or national training, accounting for 42.17%.

The question about an attitude toward the training in the COVID-19 epidemic investigation showed that over 70% of people believed that trainings was helpful. They learned about the prevention and control measures of COVID-19 and improved their skills, and the process of handling public health emergencies was standardized throughout the training. Meanwhile, 36 people (2.73%) thought that the trainings was alike, lacked innovation, and had minimal effect; 37 people thought the trainings was repetitive and tedious and did not arouse their interest in learning.

### 3.3 Ability of epidemiological investigation

#### 3.3.1 Skills for epidemiological investigation

For epidemiological investigation methods (face-to-face inquiry, telephone interview, video call, contact with other sources, etc.), 600 people chose more than three ways of investigation, accounting for 45.42%. One kind of investigation method was chosen by 241 participants.

The contents of the epidemiological investigation report were correctly selected by 828 people with 62.68% accuracy. The proportion of people who could independently write epidemiological investigation reports was 36.34% (480 subjects). A total of 494 subjects had not yet written a piece of report, accounting for 37.40%. During the epidemic, 457 people responded that their reports contained more than four items (eight items in total), accounting for 34.60%, and less than 30% (136 subjects) were able to write a complete report.

With regard to core information, 334 subjects (25.28%) could write it independently, 245 had written less than five pieces, and 10 had written more than 20 pieces. A total of 910 subjects had never written core information yet. The contents of six items of core information were chosen completely by 83 people (6.28%), and 412 people selected three or more items. A total of 905 people could correctly tell close contacts from other confusing options, with an accuracy rate of 68.51%. The scoring rate was 42.5%.

#### 3.3.2 Knowledge of site disinfection

Only 28 people (2.29%) chose the correct disinfection methods for the ground or surface of general objects in classrooms, dormitories, and dining halls. A total of 538 people were partially right. Regarding the disinfection method for patients’ blood, secretions, vomit, and other small amounts of pollutants, 775 people (40.73%) chose the correct answer, and 28 people did not know. Meanwhile, 83 people chose the correct disinfection methods for furniture, doorknobs, and household items, and 30 people did not know. The choices of 675 people were incompletely right. A total of 211 people selected the correct concept of terminal disinfection. When asked about the definition of COVID-19 cases and asymptomatic infected people, 319 answered correctly, and 29 did not know. The scoring rate was 40.4%.

#### 3.3.3 Self-evaluated data analysis ability

A total of 972 people made statistical descriptions of other disease surveillance data and wrote analysis reports, accounting for 73.58%. Among them, 428 people rated their analysis reports 7 points (median) on a scale of 10 points, 6 points for the upper quartile, and 8 points for the lower quartile. Meanwhile, 419 people could accurately describe the epidemiological analysis of the epidemic situation of infectious diseases, accounting for 31.72%, and 738 people had not written relevant analysis, accounting for 55.87%. The scoring rate was 25.67%.

#### 3.3.4 Knowledge of case reports of COVID-19

In the judgment of positive personnel, 622 people were correct, and the correct rate was 47.09%. The time limits for reporting COVID-19-positive cases during initial screening and reporting of cases and arriving at the scene after receiving the COVID-19 report were grasped by 84.41% of the subjects. The accuracy rate was low at 61.77% for the time limit of core information submission. A total of 393 people were familiar with the process of emergency events and could handle it according to the process, accounting for 29.75%. Meanwhile, 563 subjects were not familiar with any emergency response process, and 327 did not know or participate in the response. The scoring rate was 63.0%.

#### 3.3.5 Individual protection and coordination experience

In handling the epidemic, 1,317 people (99.70%) were able to wear medical gloves and masks as required, and 1,290 people (97.65%) were able to properly wear and take off protective suits. The number of staff who participated in the communication and coordination of internal departments of the local CDC, patients and close contacts, medical staff, the three-department-joint investigation and the local party committee and government was 649, accounting for 49.13%. The scoring rate was 87.25%.

#### 3.3.6 Total score

The total score ranged from 4.7 to 24.65 (the full score was 28), and the average score was 14.86 ± 3.49. The investigators who gained 60% of the full score (16.8 points) were supposed to be qualified for epidemiological investigation; that is, 395 subjects (29.9%) met the standard. The linear regression model (stepwise) showed that the significant factors of the score were epidemiological investigation experience score, education background, professional title, effect and level of training that the subjects had taken, and work experience of epidemiological investigation. One-way ANOVA and independent-sample *t*-test showed that the age group (*F* = 1.200, *p* = 0.308) and level of CDC (*t* = 1.071, *p* = 0.284) were not significant and thus not entered in the regression model. Gender was also excluded in the linear regression, although it was significant in *t*-test (*t* = 4.328, *p* < 0.01). The parameters of the regression model are shown in Table 3. ANOVA revealed that the model had statistical significance (*F* = 118.372, *p* < 0.0001). The R square of the model was 0.351.

**Table 3:** Linear regression result of total score.

Variables	Unstandardized coefficients		Standardized coefficients	*t*	Sig	Collinearity statistics	
	*B*	Std. error	Beta			Tolerance	VIF
Constant	5.330	0.454		11.733	0.000		
Epidemiological investigation experience score	1.516	0.112	0.316	13.579	0.000	0.911	1.098
Training level	0.888	0.085	0.252	10.497	0.000	0.857	1.167
Professional title	0.464	0.083	0.132	5.571	0.000	0.886	1.129
Training effect	0.609	0.104	0.136	5.864	0.000	0.920	1.088
Duration of epidemiological investigation	0.058	0.011	0.124	5.072	0.000	0.827	1.209
Education background	0.564	0.127	0.104	4.449	0.000	0.907	1.103

## 4 Discussion

This study was the first to evaluate the capability of epidemiological investigators of CDCs in Guizhou Province. Given that most of the epidemiological investigators of CDCs participated in the survey, the representativeness was acceptable. This survey was completed before October 2022, when no large-scale infection of COVID-19 was occurring in Guizhou Province (8, 9). Therefore, it presented the basic status of professional quality and response-ability of the epidemiological investigators before the large-scale outbreaks of COVID-19 infection in 2022 and also pointed out the direction for the training of epidemiologists in future. The findings revealed that epidemiological investigators were scarce in every prefecture of Guizhou Province: every prefecture had less than 13 investigators per 100,000 people on average. The majority of the subjects had a bachelor’s degree or below, and 70% of them had been involved in epidemiological investigation for less than five years. The epidemiological investigation in Guizhou’s CDCs was not technology or skill-intensive work.

The total score was not significantly different between city- and county-level CDCs; the difference brought by gender and age could be ignored. The experience of epidemiological work and training (including investigation on COVID-19 cases and contacts), educational background, and professional title were able to partially explain the outcome of the survey. The personnel structure was similar among the different levels of CDCs in Guizhou. However, using the Technique for Order Preference by Similarity to Ideal Solution, Wu Z’s research group (10) found that in Heilongjiang Province, staff from CDCs at the city level had weak judgment and assessment of an epidemic and case finding and epidemiological analysis. The main weaknesses of the staff from CDCs at the county or district level were case investigation and verification of cases, which were directly connected with the different practical works faced by the staff in CDCs at different levels (10).

This study showed that the ability of individual protection and coordination in tackling the epidemic had a high assessment score, indicating that the conceptions of self-protection and coordination in epidemic tackling were widely accepted in the fight against COVID-19. Skills were sharpened in the emergency response, as observed by Perrotta et al. (11) in their earlier study. The rate was relatively low in the part of knowledge on COVID-19 case reports. Sections such as skills for epidemiological investigation and knowledge on site disinfection had plenty of room for improvement as the average score barely reached half of the full score. However, the scoring rate of the self-evaluated data analysis ability was 25.67%, indicating the lack of self-confidence in data analysis among investigators. Li et al. (12) monograph presented a similar problem. Inexperience, low-quality data analysis required in the daily work of investigators, and various education backgrounds were considered as the reasons for the low assessment score on data analysis ability. In further training, the ability of data analysis should be improved, and many chances of data analysis being made available to epidemiological investigators, or high requirements for common reports should be proposed (13). A survey on the basic epidemiological knowledge of front-line investigators for COVID-19 (14) recorded a high awareness rate of general basic knowledge; however, a few important basic skills were not well mastered. Therefore, the application of basic knowledge in epidemiological investigation must be enhanced.

In the emergency against COVID-19, the prefectural and municipal trainings included interpretation of the latest edition of epidemiological investigation protocol, basic knowledge of COVID-19 and individual skills in practical work such as methods of disinfection and application of personal protection equipment, structures of the epidemiological report, and core information. Provincial and national training emphasized new changes in prevention and control strategies against COVID-19, organization and responsibility in epidemiological investigation, coordination and communication in emergency disposal, field epidemiology (15) and skills of data analysis. There were overlaps in all levels of trainings, but different levels of trainings were held by instructors with varying degrees of authority. It was generally accepted that provincial and national trainings enjoyed higher authority and were more systematic, while district and municipal trainings were more practical and targeted. The top-down continuing education mechanism made health professionals from different CDCs improve their professional skills and establish consistent standards to control and prevent a crisis in a relatively short time (16).

The score of each question was coded by a commonly accepted method, which assumed that every item of the questionnaire shared the same importance. As the number of items in different parts varied, the significance of comparison within different parts was limited. Training, work experience, and educational background were factors of the total score, indicating that the investigation ability could be enhanced by enriching work experience, supporting adult education, and conducting high-quality field epidemiology training programs (15, 17). In further studies, a quality assessment index system with dimension hierarchies (18) must be established, and pre-/post-evaluation design should be considered (19). In addition, self-report bias was inevitable in this questionnaire survey. For instance, in part of the efficacy of training, people seldom report negative attitudes toward training. They were prone to present positive results to show their respect to the training holders and instructors or to make themselves look like good trainees with fruitful results rather than their true feelings. The bias, to some extent, would overestimate the efficacy of training. The 10-point self-report scale on the assessment of data analysis reports would have a similar problem. More objective methods (20, 21) or indicators were recommended to analyze training effects and professional skills in future.

According to the survey of epidemiological investigators of CDCs in Guizhou Province, the demand for professional staff for the fight against COVID-19 was urgent. Qualified epidemiological investigators or public health workers with high-level professional education backgrounds were in shortage. The data analysis ability, knowledge of site disinfection, and skills for epidemiological investigation of the staff must be improved, particularly in continuing education among CDC staff. Additional chances of epidemic handling and comprehensive tasks in epidemiological investigation should be offered to inexperienced investigators.

## Data availability statement

The raw data supporting the conclusions of this article will be made available by the authors, without undue reservation. The database is available in Supplementary Table 2.

## Ethics statement

Ethical approval was not required for the study involving humans in accordance with the local legislation and institutional requirements. The studies were conducted in accordance with the local legislation and institutional requirements. The patients/participants provided their written informed consent to participate in this study.

## Author contributions

HH: Conceptualization, Data curation, Formal analysis, Investigation, Writing – original draft, Writing – review & editing. G-hY: Methodology, Supervision, Writing – review & editing. JH: Writing – review & editing. BD: Methodology, Writing – review & editing. JW: Software, Writing – review & editing. CY: Conceptualization, Investigation, Project administration, Resources, Supervision, Writing – review & editing.

## Supplementary material

SUPPLEMENTARY Table 1 Structure and scoring rule of the questionnaire.

SUPPLEMENTARY Table 2 Questionnaire database in Guizhou Province.

SUPPLEMENTARY Data sheet1 Questionnaire for epidemiological investigator.
