# Supplementary material for: Cross-sectional study on the epidemiological investigation ability of professional staff from Centers for Disease Control and Prevention in Guizhou Province
Source: Front Public Health. 2024 May 20;12:1335553. doi: 10.3389/fpubh.2024.1335553 (PMC11145900; doi:10.3389/fpubh.2024.1335553)
Supplement: SUPPLEMENTARY Data sheet1 — Questionnaire for epidemiological investigator. [file Data_Sheet_1.docx]

Survey on COVID-19 epidemic surveillance personnel in Centers for Disease Control of Guizhou Province

In order to better understand the professional level of COVID-19 epidemic investigators in Centers for Disease Control and Prevention of Guizhou Province, and make emergency preparations for the epidemic, we have designed the following questionnaire. Please fill in the questionnaire carefully according to the actual situation. Thank you for your cooperation!

1. **I Demographic Information**

Name

Gender

Age

Education Background

Professional title

Affiliation

Years of work involved in epidemiological investigation

Telephone number

1. **II Experience in handling COVID-19**

1. Business contents in charge since 2020 (multiple options available) ()

A Infectious disease prevention

B Emergency management

C Chronic disease prevention

D Tuberculosis prevention

E AIDS prevention

F Endemic disease prevention

G Prevention and control of occupational diseases

H Immunization program

I Others ()

2. Whether they have been involved in the treatment of COVID-19 epidemic since 2020 (COVID-19 positive cases and close contacts should be filled in separately)): ()

A Yes B No

If yes, the main work involved (multiple choice):

A Epidemiological investigation

B Field disinfection

C Cooperative investigation letter processing

D Information submission

E Nucleic acid collection

F Nucleic acid detection

G Quarantine point management

H Other ()

3. The number of times you have participated in the disposal of local COVID-19 epidemics since 2020 (), including the investigation and disposal of COVID-19 positive cases () and close contact investigations ().

A 0

B 1-5 times

C 6-10 times

D 11-20 times

E More than 20 times

4.Number of times you have participated in assisting COVID-19 epidemics inside and outside the province since 2020 ()

**III Ability of epidemiological**

1. When conducting epidemiological investigations of cases or close contacts, by the following means (multiple choices are available). ()

A Contact by telephone

B By videophone or video

C Face-to-face

D Ask an insider about the case or the person in close contact

E Others ()

2. What is the content of the epidemiological investigation of COVID-19? ()

A General situation

B Household income

C Onset and treatment course

D Itinerary

E Laboratory tests and

F Close contacts

H Environmental hygiene investigation of sojourn history and contact history

3. Can you independently write the core message of a positive person?

()

A Has not written

B Cannot written

C Has written since 2020

1. 4. What do you think is the core message of the COVID-19 pandemic?

5. Can you independently write an epidemiological investigation report on the COVID-19 epidemic? ()

A Can, the number (range) of copies written since 2020 includes positive case investigation reports () and close contact investigations (). B cannot C not written (skip question 6)

A 1-5

B 6-10

C 11-20

D more than 20

6. What parts do you include in your report on the epidemiology of COVID-19? (Fill in the blanks)

7. What are the close contacts of COVID-19? ()

A Living together

B Dining together

C Entertain together for a long time

D Indoor food service personnel in a confined environment or with poor ventilation

E A fellow passenger in another carriage

F The person who greeted the case from a distance outdoors

**IV On-site disinfection ability**

1. In the classroom, dormitory, canteen and other ground or general object surface for preventive disinfection methods commonly used? ()

A 500-1000mg/L chlorine disinfectant for wiping or mopping

B 250-500 mg/L chlorine containing disinfectant for wiping or mopping

C 75% alcohol to wipe or spray to disinfect

D 1000mg/L chlorine containing

disinfectant for wiping or spraying disinfection

E Don't know

2. How should small amounts of contaminants such as the patient's blood, secretions and vomit be removed? ()

A Remove with a wet rag

B Carefully remove with a gauze (or absorbent material) soaked with 75% alcohol

C Remove the gauze (or absorptive material) containing chlorine disinfectant with 500mg/L~1000 mg/L available chlorine

D Carefully remove the gauze (or absorptive material) containing chlorine disinfectant with effective chlorine of 5000mg/L~10000 mg/L

E Doesn't know

3. How to disinfect bedside tables, furniture, doorknobs and household items when they are contaminated? After removing the contaminants, ()

A Spray, wipe or soak disinfectant containing chlorine with 1000mg/L available chlorine for disinfection

B Spray, wipe or soak disinfectant containing chlorine with 500mg/L available chlorine for disinfection

C Spray, wipe or soak disinfection with chlorine dioxide disinfectant with effective chlorine 500mg/L

D the surface of the object that is not resistant to corrosion is sprayed, wiped or soaked with 2000mg/L quaternary ammonium salt disinfectant.

E Don't know

4. Are the following descriptions of terminal disinfection correct? ()

A Terminal disinfection refers to the thorough disinfection of the source of infection after it leaves the relevant place

B The objects of terminal disinfection include the pollutants discharged by cases and asymptomatic infected persons and the objects and places that may be contaminated

C Terminal disinfection does not have to carry out large area disinfection of the outdoor environment

D Places without obvious pollutants where cases and asymptomatic infected persons have been active for a short time do not need terminal disinfection

E Don't know

5. What is correct about disinfection in COVID-19 cases and asymptomatic patients? ()

A Isolation wards and transport tools of medical institutions should be disinfected at any time.

B Environmental object surface can choose chlorine disinfectant, chlorine dioxide and other disinfectants to wipe, spray or soak disinfection.

C A disinfection channel can be set up at the door of the community to spray and disinfect the personnel entering the community.

D Indoor air disinfection can choose peracetic acid, chlorine dioxide, hydrogen peroxide and other disinfectant spray disinfection.

E Don't know

**V Data analysis ability**

1. Do you carry out statistical description and write analysis reports on the data of other disease surveillance? ()

A Has written

B Has not written, skip to question 3

2. Please self-rate the quality of the monitoring data analysis report you wrote (). (Out of 10)

3. Can an accurate descriptive epidemiological analysis be carried out on the occurrence of infectious disease at the scene of the outbreak? ()

A Hasn't written B Cannot C Can

**VI. Outbreak detection and reporting**

1. Under what circumstances is it necessary to report a positive initial test for COVID-19? ()

A Single tube was positive in initial screening

B Mixed tube initial screening positive

C Mixed tube positive personnel were all found after the review of positive infection

D Recurrent infections

2. The reporting time of SARS-CoV-2 positive initial screening should be:

A Within 1 hour B Within 2 hours

C Within 4 hours D Within 12 hours

E Within 24 hours

3. How long should the epidemiological personnel arrive at the scene after receiving a report of COVID-19? ()

A Within 1 hour B Within 2 hours

C Within 4 hours D Within 12 hours

E Within 24 hours

3. How much time should the core information be submitted after the dispatcher receives the report? ()

A Within 1 hour B Within 2 hours

C Within 4 hours D Within 12 hours

E Within 24 hours

4. How much time should the dispatcher finish the report after receiving the report? ()

A Within 1 hour B Within 2 hours

C Within 4 hours D Within 12 hours

E Within 24 hours

5. How familiar are you with the emergency response process for emergencies? ()

A Did not dispose of it

B Is not aware of any requirements for the disposal process

C Needs to review the relevant documentation requirements to follow the disposal process

D Familiar with and able to handle according to the process, you are familiar with the type of incident (multiple choices) (A infectious disease epidemic, B food poisoning incident, C occupational poisoning incident, D group unexplained disease, E others ())

**VII Personal protection and communication and coordination skills**

1. Can you wear medical gloves as required in the handling of an epidemic? ()

A Yes B No

2. Can you wear a medical mask as required during the outbreak? ()

A Yes B No

3. Can you put on and take off the protective clothing correctly? ()

A Yes B No

4. Which of the following communication and coordination work have you been involved in dealing with the COVID-19 epidemic? (Multiple choice) ()

A Communication and collaboration within departments of disease control and prevention

B Communication and coordination with patients and close contacts

C Communication and coordination with medical staff

D Communication and coordination between the three public hospitals and medical staffs

E. Communicate and coordinate with local Party committees and governments

**VIII Information on participating in relevant training**

1. Have you attended any training related to the epidemiological investigation of COVID-19? ()

A Yes B No

2. What level of training have you attended? (Multiple choice) ()

A District county B City state level

C. Provincial D National

3. How effective has the training been for you? (Multiple choice)

A Learned about the COVID-19 epidemic prevention and control measures

B Improved his skills in epidemiological investigation

C Standardizes the handling process of public health emergencies

D Training is monotonous, lack of innovation, and less effective

E Repetitive training, cannot raise interest in learning

4. What type of epidemiological investigation training do you think you can most easily accept? (Multiple choice) ()

A Special lecture

B Case study

C Desktop deduction

D Epidemic response review discussion

E Practice

F Examination

G Other ()
